# Hearing characteristics of infantile-onset Pompe disease after early enzyme-replacement therapy

**DOI:** 10.1186/s13023-021-01817-1

**Published:** 2021-08-05

**Authors:** Chien-Yu Hsueh, Chii-Yuan Huang, Chia-Feng Yang, Chia-Chen Chang, Wei-Sheng Lin, Hsiu-Lien Cheng, Shang-Liang Wu, Yen-Fu Cheng, Dau-Ming Niu

**Affiliations:** 1 Department of Otorhinolaryngology—Head and Neck Surgery, Taipei Veterans General Hospital Yuli Branch, Hualien County, Taiwan; 2 Department of Otolaryngology—Head and Neck Surgery, Taipei Veterans General Hospital, Taipei, Taiwan; 3 Faculty of Medicine, National Yang Ming Chiao Tung University, Taipei, Taiwan; 4 Department of Pediatrics, Taipei Veterans General Hospital, No. 201, Sec. 2, Shipai Rd., Beitou District, Taipei City, 11217 Taiwan; 5 School of Medicine, National Yang Ming Chiao Tung University, Taipei, Taiwan; 6 Department of Speech Language Pathology and Audiology, National Taipei University of Nursing and Health Sciences, Taipei, Taiwan; 7 Department of Biomedical Engineering, National Yang Ming Chiao Tung University, Taipei, Taiwan; 8 Department of Medical Research, Taipei Veterans General Hospital, No. 201, Sec. 2, Shipai Rd., Beitou District, Taipei City, 11217 Taiwan; 9 Institute of Brain Science, National Yang Ming Chiao Tung University, Taipei, Taiwan; 10 Institute of Clinical Medicine, National Yang Ming Chiao Tung University, Taipei, Taiwan

**Keywords:** Infantile-onset Pompe disease, Enzyme-replacement therapy, Hearing loss, Congenital deafness, Lysosomal storage disorder, Glycogen storage disorder

## Abstract

**Background:**

Studies suggest that enzyme-replacement therapy (ERT) is crucial to the survival of patients with infantile-onset Pompe disease (IOPD). Hearing impairment (HI) is one of the clinical sequelae observed in long-term survivors. However, the benefits of early ERT for hearing outcomes have not yet been reported. This study aimed to investigate the impact of early ERT on IOPD patients.

**Methods:**

This retrospective longitudinal study recruited IOPD patients who were referred by newborn screening for confirmatory diagnosis based on our rapid diagnostic criteria and received early ERT treatment between January 1, 2010, and January 31, 2018. The hearing test battery included a tympanogram, otoacoustic emission, auditory brainstem evoked response (ABR), pure-tone audiometry or conditioned play audiometry.

**Results:**

Nineteen patients with IOPD were identified, 6 of whom had hearing impairment (HI); 1 had conductive HI, 2 had sensorineural HI (one had bilateral mild HI and one had mild HI in a single ear) and 1 had moderate mixed-type HI. Two patients failed the newborn screening test and had mild HI in the ABR. The mean age of the initial time to ERT was 11.05 ± 4.31 days, and the HI rate was 31.6% (6/19).

**Conclusion:**

Our study is the largest cohort to show the characteristic hearing outcomes of IOPD patients after ERT. Early ERT within 2 weeks after birth may contribute to better hearing outcomes. Clinicians should be vigilant in testing for the hearing issues associated with IOPD and should intervene early if any HI is detected.

## Introduction

Pompe disease, which is also known as glycogen storage disorder type II or acid maltase deficiency, is an autosomal recessive lysosomal storage disorder that is caused by a deficiency of the lysosomal enzyme acid alpha-glucosidase (GAA) [1–4]. The incidence of Pompe disease is variable; it occurs in an estimated 1 in 14,000 to 600,000 people worldwide, depending on ethnicity and geographic region [2, 5–10].

Pompe disease leads to the progressive accumulation of glycogen in specific organs and tissues, especially muscular (skeletal, cardiac and smooth muscles) and nervous tissues, causing abnormal motor and neural functions, including hearing loss. The presentation of glycogen storage in the organ of Corti in a knockout mouse model of Pompe disease, including in the inner and outer hair cells, the supporting cells, the stria vascularis, and the spiral ganglion neurons, suggests that this hearing impairment may be related to a cochlear pathology [11, 12].

This disease shows an extremely wide spectrum of symptom severity and is usually classified into three categories according to the time of onset: infantile-onset Pompe disease (IOPD) and late-onset Pompe disease (LOPD), and in between, non-classical infantile-onset Pompe disease [13, 14]. Patients with IOPD usually show hypotonia, muscle weakness, motor delay, feeding problems, and respiratory insufficiency right after the birth [13, 15]. Most IOPD patients experience fatal symptoms within the first year of life and cannot survive for more than two years without treatment [13, 14, 16].

Early enzyme-replacement therapy (ERT) with recombinant human alpha-glucosidase can improve the survival rate, reverse cardiomyopathy, and improve motor function in patients with Pompe disease [15–19]. Although ERT significantly increases survival, other clinical sequelae, including hearing loss and cognitive developmental delays, have been observed in long-term survivors with IOPD [15, 20]. Currently, hearing loss is recognized as an important cause of morbidity in IOPD patients. The auditory dysfunction of IOPD patients has been described in several studies; the most common type of hearing impairment is the sensorineural type [12, 13, 15, 20–23].

As in other lysosomal storage disorders, while there is a consensus in favor of early ERT before the occurrence of irreversible damage when treating IOPD [13], our previous study also showed that very early identification and initiation of ERT allows for better patient outcomes [24]. However, the benefits of early ERT in terms of hearing outcomes have not yet been reported. In this study, we investigated the hearing function of IOPD patients who received early ERT, starting around 11 days old. We also reviewed and discussed the hearing outcomes of other groups of patients who began receiving ERT at various times after birth.

## Materials and methods

### Study population

In Taiwan, Pompe disease has been included in the nationwide newborn screening program since 2008. This study included children who were referred to Taipei Veterans General Hospital (TVGH) and received a confirmed diagnosis of IOPD between January 1, 2010 and January 31, 2018. After 2010, all the referred newborns were given ERT starting within 4 h of admission if they had the following manifestations: (1) general hypotonia; (2) an elevated creatine kinase level (> 250 m/L); (3) extremely low GAA activity upon initial dried blood spot analysis (< 0.50 mmol/L/h); and (4) a high left ventricular mass index (> 80 g/m2) [24, 25]; confirmatory diagnoses using GAA gene sequencing was usually performed in 5 days passing their newborn hearing screenings, while two patients failed. The Institutional Review Board (IRB) of the Taipei Veterans General Hospital approved this study (IRB No.2020–11-004AC). All the procedures performed in studies involving human participants were in accordance with the ethical standards of the institutional and/or national research committee and with the 1964 Helsinki declaration and its later amendments or comparable ethical standards.

### Audiometric measures

Newborn hearing screening was performed by automatic auditory brainstem evoked response (aABR) after birth. Behavioral test techniques, such as conditioned play audiometry (CPA), were applied for patients under 5 years old. Pure‐tone threshold testing was performed for both air conduction and bone conduction. The average air conduction thresholds at frequencies of 500, 1,000, and 2,000 Hz were used to classify the type of hearing loss (the conductive component in case of an air‐bone gap of ≥ 15 dB) and to grade the amount of hearing loss according to the American Speech-Language-Hearing Association (ASHA) definition of the degree of hearing loss [26]. The degree of hearing loss was classified as normal (< −10 and ≤ 15 dB HL), slight (> 15 and ≤ 25 dB HL), mild (> 25 and ≤ 40 dB HL), moderate (> 40 and ≤ 55 dB HL), moderately severe (> 55 and ≤ 70 dB HL), severe (> 70 and ≤ 90 dB HL), or profound (> 90 dB HL). Two patients underwent threshold auditory brainstem evoked response (TABR) measurements when they were 0.3 and 1.1 years of age. The waveforms were analyzed by an experienced audiologist, who defined the latency of peak V. The amount of hearing loss was estimated from the detection threshold of peak V. The hearing level thresholds as estimated by TABR were categorized as normal (< 25 and ≤ 35 dB nHL), mild (> 35 and ≤ 45 dB nHL), moderate (> 45 and ≤ 65 dB nHL), severe (> 65 and ≤ 90 dB nHL), or profound (> 90 dB nHL) hearing loss [27]. Regarding the tympanometry, the tympanograms were classified as type A, As, Ad, B, or C according to Jerger (1970) [28].

### Statistical analysis

The quantitative data were summarized as the means ± standard deviation (SD), and the categorical variables were summarized as percentages. All the statistical analyses were performed using IBM SPSS 20.0 software (IBM Corp., Armonk, NY, USA).

## Results

### Subjects

Nineteen patients with IOPD were included in the current study. Table 1 summarizes the demographic features of each of the patients. All the patients received their first ERT within 4 h of admission at a mean age of 11.05 days (range 6–23 days).

**Table 1:** Patient characteristics of 19 patients with IOPD

Patient no. (n = 19)	Sex	GA, week, BBW (kg)	Age at referral (day)	Age at first ERT (day)	End of study age (year)	GAA mutation
1	F	37, 3.2	18	18	9.5	c.1411_1414del, (E471fsX5), heterozygous c.872T → C, (p.L291P) heterozygous
2	M	38, 3.5	15	15	8.4	c.1935 C → A, (p.D645E), homozygous c.1726 G → A, (p.G576S), homozygous
3	M	39, 3.3	9	9	8.4	c.1935 C → A, (p.D645E), heterozygous c.2303 C → T, (p.P768L), heterozygous
4	M	38, 3.1	12	12	8	c.1396 G → T, (p.V466F), heterozygous c.1935 C → A, (pD645E), heterozygous
5	F	39, 3.0	9	9	7.5	c.1935 C → A, (p.D645E), homozygous c.1726 G → A, (p.G576S), homozygous
6	F	39, 3.1	8	23	6.5	c.2238 G → C, (p.W746C), heterozygous c.2237 G → A, (p.W746X), heterozygous c.1726 G → A, (p.G576S), heterozygous
7	F	34, 2.2	12	12	6.5	c.1935 C → A, (pD645E), heterozygous IVS7 + 2T → C, heterozygous c.1726 G → A, (p.G576S), heterozygous
8	M	39, 3.7	7	7	6.4	IVS7 + 2T → C, heterozygous c.1935 C → A, (p.D645E), heterozygous c.1726 G → A, (p.G576S), heterozygous
9	F	39, 2.9	13	13	6	c.1082 C → T, (p.P361L), heterozygous c.1935 C → A, (p.D645E), heterozygous c.1726 G → A, (p.G576S), heterozygous
10	F	39, 2.9	10	10	5.7	c.1935 C → A, (p.D645E), homozygous c.1726 G → A, (p.G576S), homozygous
11	F	41, 2.6	6	6	5.6	c.1935 C → A, (p.D645E), homozygous c.1726 G → A, (p.G576S), homozygous
12	F	36, 3.3	8	8	4.8	c.1411_1414del, (E471fsX5), heterozygous c.1935 C → A, (pD645E), heterozygous c.1726 G → A, (p.G576S), heterozygous
13	M	39, 2.5	13	13	4.6	c.1726 G → A, (p.G576S), heterozygous c.1935C → A, (pD645E), heterozygous c.2274insC, (p.G759fs), heterozygous
14	F	39, 3.1	7	8	2.4	c.1935 C → A, (p.D645E), homozygous c.1726 G → A, (p.G576S), homozygous
15	F	38, 3.5	7	9	3.1	c.1411_1414del, (E471fsX5), heterozygous c.752C > T;c.761 C > T,p.S251L; S254L c.1843 G > A,p.G615R
16	F	37, 3.1	9	10	3	c.1636 + 10 C > T c.2024_2026del,p.N675del c.241 C > T,p.Q81*
17	F	38, 3.6	14	14	2.8	c.2228 A > C,p.Q743P c.1726 G → A, (p.G576S), heterozygous c.1935C → A, (pD645E), heterozygous
18	M	39, 3.1	6	6	2.5	c.2238 G → C, (p.W746C), heterozygous c.1726 G → A, (p.G576S), heterozygous c.1935C → A, (pD645E), heterozygous
19	M	38, 3.8	7	8	2	c.1935 C → A, (p.D645E), homozygous c.1726 G → A, (p.G576S), homozygous
Mean (SD)			10.0 (3.42)	11.05 (4.31)	5.46 (2.33)	

F, female; M, male; GA, gestational; BBW, birth body weight; and SD: standard deviation

### Characteristics of hearing loss

Table 2 summarizes the auditory test results of the patients. All the patients except one underwent newborn hearing screening at birth; sixteen patients were recorded as having normal values for the hearing screenings, two patients had hearing impairment, and one patient was not screened for hearing. The ages of the patients at their last hearing tests ranged from 2 to 9.5 years old (5.46 ± 2.33). At the most recent assessment, pure-tone audiometry (PTA) or conditioned behavioral audiometry showed that 1 child had a mild unilateral hearing impairment (33 dB) and 1 had a moderate mixed-type hearing impairment according to the ASHA hearing classification system. Two patients had conductive hearing loss with an air-bone conduction gap of 15 dB, which was suggestive of chronic middle ear dysfunction. The TABR showed that 2 patients had mild bilateral hearing loss (35–40 dB nHL).

**Table 2:** Summary of audiometric results for the 19 IOPD patients at birth and after enzyme replacement therapy

Patient no. (n = 19)	Hearing screen (T-ABR) right/left (dB)	OAE	Age of last hearing exam (year)	Average hearing threshold (dB) (500, 1000, 2000 Hz)		Tym (right/left)		Post-ERT hearing loss type^a, b^
				Right	Left	Right	Left	
1	Pass	+	7.5	21.7	21.7	A	A	Normal
2	NA	+	6.7	25.0	33.3	A	A	Single ear SNHL, Mild
3	Pass	−	6.8	41.7	45.0	C	C	Bil. mixed HL, Moderate
4	Pass	+	1.2	21.7	21.7	A	A	Normal
5	Pass	+	5.5	16.7	15.0	A	A	Normal
6	Pass	+	5.4	16.7	15.0	A	A	Normal
7	Pass	+	4.7	13.3	10.0	A	A	Normal
8	Pass	+	4.8	20.0	13.3	A	A	Normal
19	Pass	+	4.9	25.0	23.3	A	A	Normal
10	Pass	NA	4.3	26.7	28.3	B	B	Bil. cond. HL, Mild
11	Pass	NA	4.7	26.7	26.7	A	A	Bil. SNHL, Mild,
12	Pass	+	3.1	15.0	15.0	A	A	Normal
13	Pass	+	2.8	20.0	20.0	A	A	Normal
14	Pass	+	1.1	25.0	25.0	A	A	Normal
15	Failed	−	1.1	35.0	35.0	A	A	Bil. SNHL, Mild
16	Pass	+	1.8	20.0	20.0	A	A	Normal
17	Pass	+	1.5	20.0	20.0	A	A	Normal
18	Pass	+	0.8	20.0	20.0	A	A	Normal
19	Failed	+	0.3	40.0	40.0	A	A	Bil. SNHL, Mild

T-ABR, threshold auditory brainstem evoked response; OAE, otoacoustic emission; Tym., tympanogram; HL, hearing loss; cond., conductive; SNHL, sensorineural hearing loss; bil., bilateral; ABG, air-bone conduction gap; + , present; -, absent; and NA, not applicable

^a^ASHA definition of the degree of hearing loss (Clark 1981): The hearing loss degree was classified as normal (< -10 ≦ 15 dB HL), slight (> 15 ≦ 25 dB HL), mild (> 25 ≦ 40 dB HL), moderate (> 40 ≦ 55 dB HL), moderate severe (> 55 ≦ 70 dB HL), severe (> 70 ≦ 90 dB HL), or profound (> 90 dB HL) hearing loss

^b^Conductive component in case of an air‐bone gap of ≥ 15 dB

## Discussion

In this study, we reported the hearing test results of the IOPD patients, and we found that the patients in our cohort had early ERT initiation time and a lower hearing impairment rate (with an average ERT initiation time of 11.05 days and 31.6% hearing impairment in the last evaluation after treatment). To our knowledge, this is the largest cohort of patients to be investigated for the audiological characteristics of IOPD after receiving early ERT.

Hearing loss in IOPD may be sensorineural, conductive, or a mixed type. In the present study, sensorineural hearing loss is the most common type, which is consistent with earlier studies [12, 22]. Martin et al. first recognized both central and peripheral nervous system dysfunctions caused by glycogen storage through anatomopathological study [29]. Kamphoven et al. first reported their inference about inner ear involvement via otoacoustic change in IOPD patients in 2004. They found abnormal glycogen storage in spiral ganglion cells, supporting cells, stria vascularis, and both the inner and outer hair cells of the cochleas in GAA knockout mice, illustrating the possible mechanism of inner ear involvement [12, 30]. Kishnani et al. also found missing OAE and aberrant wave latencies for the ABR in some patients with Pompe disease [13]. In our cohort, 5 of 6 hearing loss patients had sensorineural hearing loss, two of whom failed the newborn hearing screening after birth. Case 3 did not receive a newborn hearing screening due to a personal reason and had single-ear sensorineural hearing loss upon follow-up. The cases who were congenitally hearing impaired may be related to Pompe disease.

Conductive hearing loss can also be observed in patients with Pompe disease [12, 15, 21, 31]. GAA deficiency causes an abnormal accumulation of lysosomal glycogen in many cell types, most notably in skeletal muscle, leading to cell and tissue dysfunction. Even with ERT, which reverses the myopathy of the respiratory and cardiac muscle tissue, some phenotypes such as ptosis, dysphagia and speech disorders may still occur, probably due to the unresponsiveness of the responsible small muscles [32]. Otitis media with effusion is commonly seen in IOPD and is the major cause of conductive hearing loss. Van Capelle et al. reported on 11 IOPD patients who received ERT; after 1–6 years of ERT, 90.9% of them had hearing impairment, and 75% of all hearing tests showed conductive hearing loss [22]. They inferred that the cause of mixed hearing loss was the accumulation of glycogen in the tensor veli palatini muscle during gestation. Rohrbach et al. reported one IOPD patient who received ERT at the age of 8 weeks and responded as type B to tympanometry when followed up at the age of 8 months, improving in behavioral audiometry after the paracentesis of both ears [21]. Chien et al. reported that 60% of their IOPD patients who received ERT at an average age of 21.60 days (6–34 days) had hearing impairments, including 4 conductive types and 2 mixed types. In addition, 5 of the 6 children received ventilation tube insertion as a primary treatment [15, 31]. In our cohort, only 10.5% (2 of 19 cases) of the patients were found to have conductive hearing loss, and none of them received ventilation tubes. The relatively low rate of conductive hearing impairment was probably due to the effect of early ERT, which prevented or decelerated the dysfunction of the Eustachian tubes or other middle ear muscles.

The natural course of IOPD without ERT would be progressive cardiomyopathy and generalized myopathy or muscular hypotonia [33]. Furthermore, most IOPD patients would suffer from disease progression leading to respiratory failure within the first year of life [2, 16]. ERT, however, not only enhances overall survival [4, 15, 20, 22] but also improves motor and cognitive functions in patients with IOPD [25, 34]. Our previous studies also showed that early ERT can preserve the long-term outcomes of patients with IOPD, including their motor and cognitive functions [25, 35]. While most studies showed dissatisfactory hearing results even after ERT, we reported better hearing performance in our cohort. Previous TVGH data reported by Yang et al. in 2016 showed normal hearing results in 14 patients [25]. By including and following those 14 patients with a thorough hearing test battery and adding 5 additional patients to the current cohort, we found that 2 children developed sensorineural hearing loss, 3 developed conductive hearing loss, and 1 developed mixed hearing loss. The better hearing outcomes reported in our cohort indicate that early ERT could have a positive effect not only on the development of the cochlear and nervous systems but also on the muscular components associated with Eustachian tube or middle ear function.

Although impaired hearing function has been reported in patients with IOPD, many studies mentioned neither the test battery used for the hearing evaluations nor the criteria used to report the degree of hearing loss, thus making it difficult to compare the results among different groups (Table 3). Hahn et al. in 2015 reported 23 cases of IOPD but mentioned only 3 cases with hearing results [23]. Our study is the largest study (19 cases) to reinforce the detailed information on hearing impairment by performing multiple batteries of hearing examinations, including tympanograms, OAE, ABR, PTA and CPA. We also graded the severity of hearing loss according to the ASHA definition of hearing impairment. Through integrations with adequate hearing tools and grading systems, we may obtain more information on the effect of ERT through longitudinal studies.

**Table 3:** Hearing outcomes among IOPD studies

Authors	Year	Country	Patient no.	Gender		Age of ERT (day)	Hearing exam tool	Hearing outcome	Hearing impairment no. (ratio)
				Male	Female				
Kamphoven [12]	2004	Netherlands	4	NA	NA	NA	Tym, OAE, ABR, PTA	4 hearing deficits (30–70 dB)	4 (4/4)
Kishnani [13]	2006	USA	8	4	4	184.13	OAE, ABR	5 hearing deficits	5 (5/8)
Rohrbach [21]	2010	Switzerland	1	0	1	56.00	Tym, OAE, ABR, CPA	Mixed	1 (1/1)
van Capelle [20]	2010	Netherlands	11	5	6	87.55	Tym, OAE, ABR	10 hearing deficits (30–90 dB)	10 (10/11)
Ebbink [20]	2012	Netherlands	10	5	5	91.80	NA	9 hearing deficits (30–90 dB)	9 (9/10)
Hahn [23]	2015	Germany	23	13	10	103.83	NA	43% deceased 3 hearing impairment	3(NA^a^)
Chien^15^	2015	Taiwan	10	NA	NA	21.60	NA	4 conductive, 2 mixed	6 (6/10)
Hsueh	2021	Taiwan	19	6	13	11.05	Tym, OAE, ABR, PTA, CPA	2 conductive, 1 mixed, 4 sensorineural	6 (6/19)

ABR, auditory brainstem evoked response; Tym, tympanogram; OAE, otoacoustic emission; PTA, pure tone audiometry; CPA, conditioned play audiometry; and NA, not applicable

^a^Not mentioned in the article (43% of 23 cases deceased)

It is difficult to compare our outcomes with those of other groups due to a lack of detailed hearing data from their studies. Several reasons may underlie the better hearing outcomes obtained in the current study. The racial difference should be considered first. The cohorts of both Chien et al. and our study are from Asian populations [15], and the different genetic background from the subjects of most other studies may contribute to different results. In addition, the large-scale newborn screening program and national insurance coverage of enzyme replacement therapy for Pompe disease in Taiwan may promote earlier initiation of treatment and better hearing outcomes. By combining the 2 cohorts from Taiwan, we propose that starting ERT early after birth can lead to better hearing outcomes than late treatment. Additionally, the lower rate of hearing impairment (31.6%) in our cohort (ERT starting at 11.05 days after birth) group than in Chien’s study [15] (60%, 20.60 days after birth) further suggests the benefit of early ERT within two weeks for the IOPD patients. And, after 10 years treatment, no IOPD patients in our series needed any kind of hearing aids. Our study may enhance awareness of early intervention before hearing-related morbidities can develop in patients with IOPD.

Our study has several limitations. First, since Pompe disease is rare, a small sample size is an inevitable issue. In addition, ERT was first approved for Pompe disease treatment in Taiwan in 2006, and all the follow-up instances have taken place since then, limiting the length of the follow-up period to date. However, our study is one of the largest cohorts to provide data on both early ERT treatment and hearing examination results. Third, there is a paucity of direct evidence to show whether better hearing results after ERT are due to reduced glycogen accumulation in the auditory system. In fact, the blood-labyrinth barrier, which is similar to the blood–brain barrier, may limit the effects of the recombinant enzymes used for ERT [36–38]. Further pathological or radiological studies may be required to study the mechanism of hearing preservation in the future. Last, control group patients are difficult to include because ERT is covered by the National Health Insurance, and early ERT treatment has been the gold standard in Taiwan.

## Conclusions

Our study is the largest cohort to provide detailed data of hearing outcomes in IOPD patients who received early ERT treatment. Early diagnosis and early initiation of ERT within two weeks after birth appear to contribute to improved hearing outcomes for patients with IOPD. Clinicians should be vigilant for the hearing issues associated with IOPD and intervene early if any hearing impairment occurs in their IOPD patients.

## Data Availability

Detailed data can be found in the “Methods” section.
